# Serious Games Based on Cognitive Bias Modification and Learned Helplessness Paradigms for the Treatment of Depression: Design and Acceptability Study

**DOI:** 10.2196/37105

**Published:** 2023-05-03

**Authors:** Arka Ghosh, Jagriti Agnihotri, Sradha Bhalotia, Bharat Kumar Sati, Latika Agarwal, Akash A, Swastika Tandon, Komal Meena, Shreyash Raj, Yatin Azad, Silky Gupta, Nitin Gupta

**Affiliations:** 1 Department of Biological Sciences and Bioengineering Indian Institute of Technology Kanpur Kanpur India; 2 Department of Cognitive Science Indian Institute of Technology Kanpur Kanpur India; 3 Mehta Family Center for Engineering in Medicine Indian Institute of Technology Kanpur Kanpur India

**Keywords:** serious games, cognitive bias modification, learned helplessness, depression, digital intervention, mobile phone

## Abstract

**Background:**

Depression is a debilitating mental health disorder, with a large treatment gap. Recent years have seen a surge in digital interventions to bridge this treatment gap. Most of these interventions are based on computerized cognitive behavioral therapy. Despite the efficacy of computerized cognitive behavioral therapy–based interventions, their uptake is low and dropout rates are high. Cognitive bias modification (CBM) paradigms provide a complementary approach to digital interventions for depression. However, interventions based on CBM paradigms have been reported to be repetitive and boring.

**Objective:**

In this paper, we described the conceptualization, design, and acceptability of serious games based on CBM paradigms and the learned helplessness paradigm.

**Methods:**

We searched the literature for CBM paradigms that were shown to be effective in reducing depressive symptoms. For each of the CBM paradigms, we ideated how to create a game so that the gameplay was engaging while the active therapeutic component remained unchanged.

**Results:**

We developed 5 serious games based on the CBM paradigms and the learned helplessness paradigm. The games include various core elements of gamification, such as goals, challenges, feedback, rewards, progress, and fun. Overall, the games received positive acceptability ratings from 15 users.

**Conclusions:**

These games may help improve the effectiveness and engagement levels of computerized interventions for depression.

## Introduction

### Background

Depressive disorders are a leading cause of disability worldwide, affecting >264 million people [1]. Antidepressant medications and cognitive behavioral therapy (CBT) are currently the most effective treatment modalities for depression [2-4], but there is a large treatment gap owing to the lack of accessibility, high cost of therapy, and social stigma associated with the visits to a psychiatric clinic [5-7]. In the past 2 decades, digital interventions have been gaining traction. The most widely studied digital intervention for depression is computerized CBT (cCBT). Although cCBT is an effective form of treatment [8-10], a recent meta-analysis has demonstrated that guided cCBT is less acceptable than being on a waiting list [11]. There is a need for other digital treatment modalities.

The cognitive theory of depression posits that information processing—attention, interpretation, and memory—is negatively biased in depression [12,13]. This biased information processing has been implicated in the onset and maintenance of depression [14]. Experimental studies have corroborated the cognitive model by providing empirical support for self-referential information processing, attention bias, interpretation bias, and memory bias [15-19]. Psychological training paradigms, known as cognitive bias modification (CBM) paradigms, have been used to modify these cognitive biases in subthreshold, clinical, and remitted populations [20-28], albeit with mixed results. Despite the mixed results and small effect sizes [29,30], this field promises novel treatment approaches and warrants further research.

The previously mentioned evaluations of CBM paradigms have been mostly performed in laboratory settings; these paradigms have received much less attention than cCBT as a potential treatment approach for depression outside the laboratory [31-33]. CBM paradigms tend to have low user engagement owing to the repetitiveness of the paradigms [34-37] and a lack of credibility in some cases [34,38]. In a digital intervention, in which the user needs to engage with a paradigm without any external supervision, the paradigm’s ability to engage becomes the determining factor for the success of the intervention.

One way to make the CBM paradigms more engaging to users is by developing serious games based on the paradigms [39]. A game serves as an interactive medium that provides game designers with considerable control in creating engaging experiences and can reduce the attrition rate in digital interventions [40]. Serious games have been evaluated for multiple conditions, including posttraumatic stress disorder [41,42], autism spectrum disorder [43-45], attention-deficit/hyperactivity disorder [46], cognitive functioning [47,48], alcohol use disorder [49,50], trait anxiety [51,52], etc. The results of a meta-analysis that evaluated the effectiveness of serious games demonstrated a moderate effect size of serious games for reducing psychiatric disorder–related symptoms compared with no intervention controls [53]. In a review of 4 studies that evaluated CBM interventions based on serious games, only 2 studies reported their gamified interventions to be effective [54].

Serious games have been developed for various aspects of depression. Bespoke serious games have been created to diagnose depression [55,56], treat depression [57-63], and describe the experience of depression either metaphorically [64] or literally [65]. Off-the-shelf commercial games have also been used to target depression [66]. There are multiple serious games based on different therapeutic techniques—CBT [67-69], solution-focused therapy [70], and interpersonal therapy [68]—that do not target depression specifically but can be useful. There is only 1 serious game based on a CBM paradigm targeting depression, but it was found to be ineffective [62]. Serious game–based interventions have been shown to be acceptable to the users overall [57,60,67-69]. A recent review, however, suggests that the effectiveness and acceptability data are not convincing enough yet to warrant clinical adoption [71].

### Objective

In this study, we selected specific CBM paradigms that had received empirical support for targeting cognitive biases underlying depression and appeared suitable for conversion to games. Then, we described the design and development of these games in detail. Furthermore, we developed 1 serious game based on the learned helplessness theory.

## Methods

### Embedding Training Paradigms Into Serious Games

We searched the literature for known CBM paradigms and selected those that have shown promise in reducing depressive symptoms and were amenable to conversion to games. Our team discussed multiple game ideas for each paradigm. While thinking of the ideas, the primary aim was to make the gameplay engaging while keeping the active therapeutic component unchanged. After multiple sessions on ideations, the team selected the most promising game idea for each paradigm. Once the team agreed on a game idea, minimally viable prototypes were created and tested within the team. These minimally viable prototypes were improved upon to create the final versions by using the feedback obtained from informal tests performed in a larger group of approximately 10 friends and colleagues.

### Software Development

We used Angular 8 (Google LLC) and JavaScript (ECMAScript 2018) for creating the front end of the games and Django-rest-framework 3.9.2 (Encode OSS Ltd) and Django 2.2 (Django Software Foundation) for creating the backend of the games. Two games required the detection of the swipe gesture on the screen, for which we used a readily available library, *hammer.js*. For 1 game that required the simulation of gravity and collision in the game environment, we used the *phaser.js* framework. The games were optimized for smartphone screens and were also compatible with tablets, laptops, and desktop devices. We created the graphical elements required for the game using Adobe Illustrator (Adobe Inc). Musical elements required for some of the games were collected from open web resources. The *negative attention bias training game* required the combination of 20 rhymes (Rock a bye baby, Wheels on the bus, etc) and famous compositions (Ode to joy, Für Elise, etc). The musical notations for these rhymes and compositions were collected from various YouTube channels. A few of them were readily available in the scientific pitch notation. For the others, we deduced the musical notations from the simple piano versions of these rhymes and compositions available on YouTube.

### Feedback on the Games

To obtain a preliminary sense of the acceptability of the games, we asked 15 pilot users (n=8, 53% male and n=7, 47% female) to provide feedback on the 3 aspects of the games. The users were recruited from the Indian Institute of Technology, Kanpur community, by word of mouth. Potential users who expressed interest were sent an email detailing the procedure to participate in the study and to provide feedback. They were also sent an informed consent form via email. They were required to include their name and signature in the informed consent form and email it back to the study team. These users rated the games on three aspects: (1) *The instructions on how to play the game were clear*, (2) *The game was fun to play*, and (3) *The purpose of the game was clear*. The users rated these aspects on a 5-point scale—(1) strongly agree, (2) agree, (3) neutral, (4) disagree, and (5) strongly disagree. These ratings were mapped to scores of 2, 1, 0, −1, and −2, respectively. For the game for learned helplessness, we asked the users an additional question, *In this game, you were presented with some unsolvable puzzles and some solvable puzzles. After you completed the game, the logic of the game was presented to you. After playing the game and reading the explanations, which of the following statements are true (tick all that apply)*?

The following options were provided: (1) *I have an understanding of learned helplessness*; (2) *I felt angry or frustrated while I was stuck with the puzzles*; (3) *After the logic of the game was explained, my frustration or anger reduced*; and (4) *After the logic of the game was explained, my frustration or anger did not reduce*. The feedback was collected as a preliminary pilot for a larger study.

### Ethics Approval

Ethical clearance was provided by the Institutional Ethics Committee of the Indian Institute of Technology Kanpur (IITK/IEC/2019-20/II/4).

### Analysis

We calculated the average value of the feedback received on the different aspects of the game. We also checked whether the feedback received was significantly different from 0 using the Wilcoxon signed rank test. Python (version 3.10.5; Python Software Foundation) and MATLAB R2020b (Mathworks) were used for data analysis.

## Results

### Design Philosophy

Our main design objectives were to make the games effective by using evidence-based paradigms and engaging by incorporating 6 out of the 7 core elements of gamification. The evidence-based paradigm used for the individual games is described in individual sections throughout the paper. CBM paradigms have been reported to be boring and repetitive [34-37]. We hypothesized that making the games fun and engaging would increase the likelihood of spontaneous gameplay and, consequently, increase the CBM dosage without external supervision. Studies have reported that the rationale behind some CBM interventions is not apparent to the users, which can contribute to the lack of engagement [34,38]. To tackle this, we added a brief section called *Science behind the game* to each game.

Individuals experiencing an episode of depression have decreased cognitive abilities [72]. Considering this, we designed the gameplay to be easy and intuitive, such that anyone with some experience in using smartphone apps and a basic familiarity with the English language could play the games. We also kept the hardware requirements for the games minimal so that they could be played on low-end smartphones. At the beginning of the games, we gave simple in-game instructions or in-game tutorials. To avoid an artificial limit on the practice of CBM, we designed the games to be never-ending (except for the game for learned helplessness, in which the design required a definite ending). The difficulty level of each game adapted to the skill level of the user to create the *flow* experience [73]. Apart from making the games intrinsically engaging, we also added some extrinsic motivators to play the games, such as badges and scores, or the sensory experience of a familiar musical tune. The badges served as short-term rewards for playing the games, and the scores served as in-game currency, which could be exchanged for lives or hints in the games. We describe how our games are based on the elements of gamification in the later section titled, *How game-like are our games?*

### Game for Automatic Interpretation Bias Modification

People with depression have an automatic negative interpretation bias [74]. Cowden Hindash et al [21,75,76] used a modified word sentence association paradigm for participants with dysphoria to confirm and modify the automatic negative interpretation bias. The bias modification paradigm also showed a near-transfer effect on the Scrambled Sentence Task [77] and a far-transfer effect of increased resiliency on a laboratory stressor [21].

**Figure 1:**
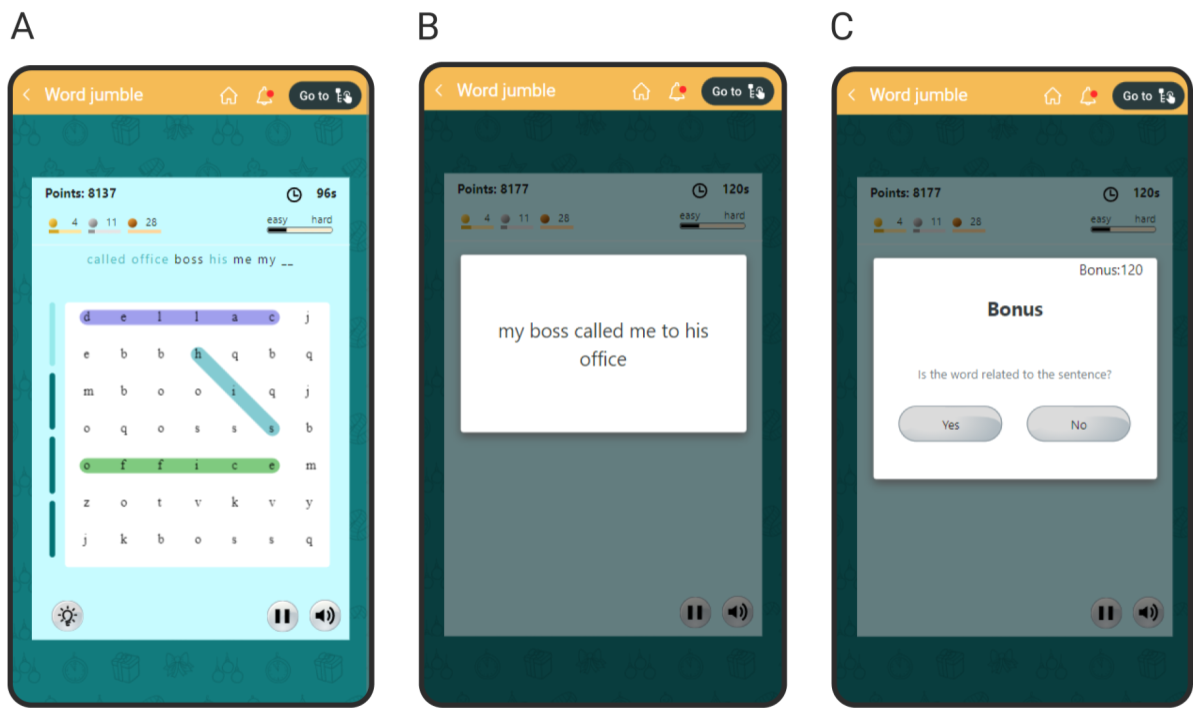
Screenshots of the game for automatic interpretation bias modification. (A) The user finds words that are contained in the sentence by linearly connecting letters in the grid. (B) The ambiguous sentence is shown in full once the user finds 4 words. (C) The user is asked if the word (*Bonus* in the shown example) is related to the sentence on the previous screen.

In the task of modified word sentence association paradigm for participants with dysphoria, a self-relevant ambiguous sentence (*My mom called me to tell me the news*) is shown for a short duration, followed by a positive or negative word. The user is asked whether the word is related to the sentence. The user can answer *Yes* (related) or *No* (unrelated). The user is trained, via text-based feedback, to associate positive words with ambiguous sentences; the feedback is *Correct* if the answer is *Yes* for a positive word or *No* for a negative word; otherwise, the feedback is *Incorrect*. We converted this paradigm into a serious game in the following manner: The game displays an alphabet grid. The user’s goal is to find the words (that can be formed by connecting letters in the grid; Figure 1A) that form the ambiguous sentence. The game screen includes several empty bars, equal to the number of words to be found. As the user finds a word, a bar gets filled to provide the users with a sense of progress in the game and indicate the number of words found. Once the user finds 50% of the words, we reveal the full ambiguous sentence (Figure 1B), and then we show a positive or negative word and ask the user if the word and the sentence are related (Figure 1C). The user can answer by clicking on *Yes* (related) or *No* (unrelated) buttons. Quicker answers are given higher rewards to encourage the users to answer with the first thought they have. An incorrect answer receives 0 points. To prevent the users from clicking *Yes* for all positive words and *No* for all negative words, we used decoy sentence-word pairs, in which the sentence was unambiguous, and the negative word was related, while the positive word was unrelated [78].

The users earn reward points by finding words in the grid. Valid English words that are not parts of the sentence also earn points. The points, apart from being a short-term reward, also serve as an in-game currency to buy extra time or hints. Apart from points, the users also receive other short-term rewards in the form of bronze, silver, and gold badges for completing 4, 10, and 26 levels, respectively.

The game adapts to the user’s level of competence by varying the number of words hidden and allotted time per difficulty level (Figure S1 in Multimedia Appendix 1). To help the user in learning to play the game, the initial 4 levels of the game keep the words of the sentence fully visible in a jumbled order and give the user 150 seconds (in the first 2 levels) or 120 seconds (in the next 2 levels) to find the words in the grid. A bar, ranging from *Easy* to *Hard*, indicates the game’s current difficulty level.

There are 42 levels (sentence-word pairs) in the game. The difficulty of each level depends on the number of words to be found and the complexity of the grid. These, in turn, depend on the length of the sentence and the length of the longest word. The levels are arranged to gradually increase the difficulty level. After the completion of all the levels, the game loops back to the first level.

### Game for Executive Control Training

**Figure 2:**
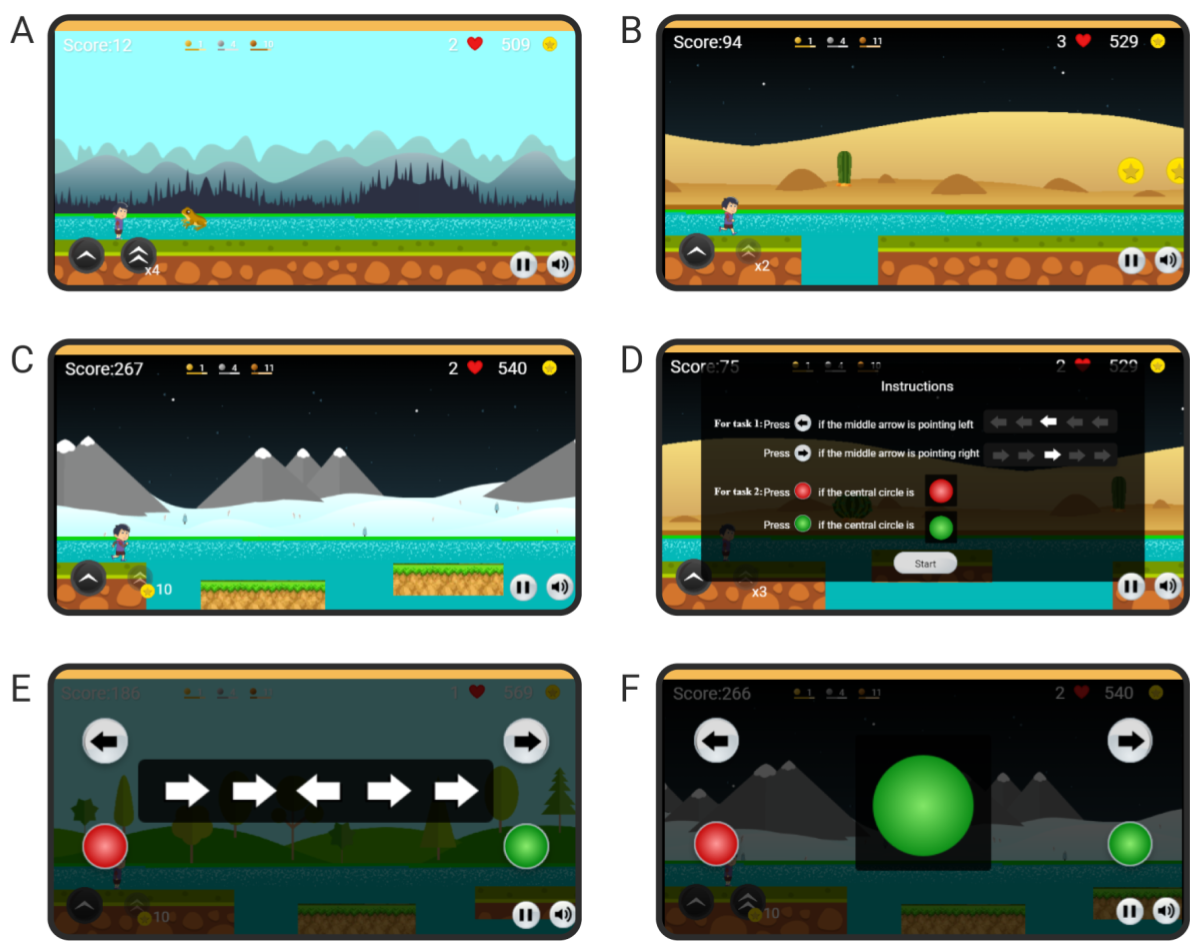
Screenshots of the game for executive control training. (A) The avatar faces an obstacle, a jumping frog. (B) The avatar faces an obstacle, a pit. (C) The avatar faces a longer pit with floating platforms. (D) The instructions for the cognitive bias modification paradigm. (E) An incongruent flanker task as a part of the cognitive bias modification paradigm. The user must press the button corresponding to the middle arrow in the flanker task. (F) A green circle as a part of the cognitive bias modification paradigm. The user must press the button (green button on the right side) corresponding to the circle in the center.

Rumination, a core factor in depression, affects the duration [79] and intensity [80] of depressive episodes. Multiple studies have established that depressive rumination is associated with an inability to inhibit the processing of emotional information [81,82]. Executive control is one mechanism that regulates the ability to inhibit emotional information in healthy individuals [83-85]. Cohen et al [86] hypothesized that the difficulty in using executive control might be the factor behind impaired inhibition in ruminators. Cohen et al [86] designed a paradigm to train individuals to exert executive control before exposure to an emotional stimulus, followed by a discrimination task. They observed that individuals trained with this paradigm were less likely to engage in a state of rumination [86] and more likely to use reappraisal—an effective emotion regulation technique—more frequently and efficiently [87]. On the basis of this executive control training paradigm [87], we designed a 2D platform game to resemble the classic video game, Super Mario Bros. (Figure 2). The goal of the user is to keep a running avatar alive by jumping over obstacles (sitting or jumping frogs; Figure 2A) or pits (Figure 2B). The pits are of different types: some requiring a single jump, some requiring a double jump, and some including floating platforms in between (Figure 2C). The floating platforms are also of 2 kinds—some are stationary when the avatar lands on them, whereas others start to fall upon the landing of the avatar. The avatar sometimes passes through an underground tunnel, and on returning to the ground level, the background landscape changes to maintain a diversity of visuals. An in-game tutorial is shown to make the user familiar with the game’s rules and controls. On a keyboard-based device, the arrow keys can be used to play the game. On a touchscreen device, buttons corresponding to the arrow keys are shown on the screen itself.

As time progresses, the speed of the avatar increases to increase the difficulty level. The score in the game increases over time as the avatar continues to run. Intermittently, the game screen presents coins, which can be collected by jumping. The coins serve as an in-game currency. Initially, the user is given 3 lives and 5 double jumps for free. Once the user exhausts the free double jumps, each double jump costs 10 coins. In addition, once the user exhausts the free lives, additional lives can be purchased with coins with gradually increasing difficulty: the first purchase costs 10 coins and each purchase after that costs twice the amount of the last purchase.

At predetermined points in the game, the avatar stops in front of a large pit with floating platforms. At this point, the executive control training task is shown to the user (Figure 2D). An incongruent or a congruent set of flanker arrows (Figure 2E) is shown with a 50:50 probability. The user is required to press an arrow key corresponding to the middle arrow in the set. The flanker arrows remain visible until the user responds or for a maximum of 1000 milliseconds. After the flanker set disappears, an image is shown for 100 milliseconds. The image is neutral or negative with an 80:20 probability following a congruent set and with a 20:80 probability following an incongruent set. After a further gap of 50 milliseconds, during which no stimulus is shown, a green or red circle is shown with a 50:50 probability (Figure 2F). The user is required to respond according to the color of the circle (up arrow for green and down arrow for red). The circle remains visible until the user responds or for a maximum of 2000 milliseconds (Figure S2 in Multimedia Appendix 1). These 3 steps—including the arrows, the image, and the circle—constitute 1 round of training. The next round of similar training steps begins after a delay of 2000 milliseconds. After 3 rounds of training, the gameplay begins again and continues for approximately 30 seconds before the next batch of training begins. The instructions for choosing the correct keys are shown before each round of training. If the user presses the correct keys for both the flanker task and the colored circle task in a round, it is considered a correct response. The users are incentivized to perform the task diligently, as correct choices reward them with double jumps or additional lives. The users can also earn short-term rewards in the form of bronze, silver, and gold badges for 6, 15, and 39 correct responses, respectively.

### Game for Negative Attention Bias Modification

The vulnerability model of low self-esteem and depression states that low self-esteem increases the risk of future depressive episodes [88,89]. Accordingly, improving one’s self-esteem should reduce the risk of depression. Individuals with lower self-esteem are more attentive to rejection cues, which in turn increases their tendency to interpret more and more social cues as rejecting, thus perpetuating the cycle of vigilance and low self-esteem [90,91]. Dandeneau et al [92] used an attention-training paradigm to modify this negative attention bias and observed that it led to a more resilient self-esteem against a laboratory-based rejection manipulation.

**Figure 3:**
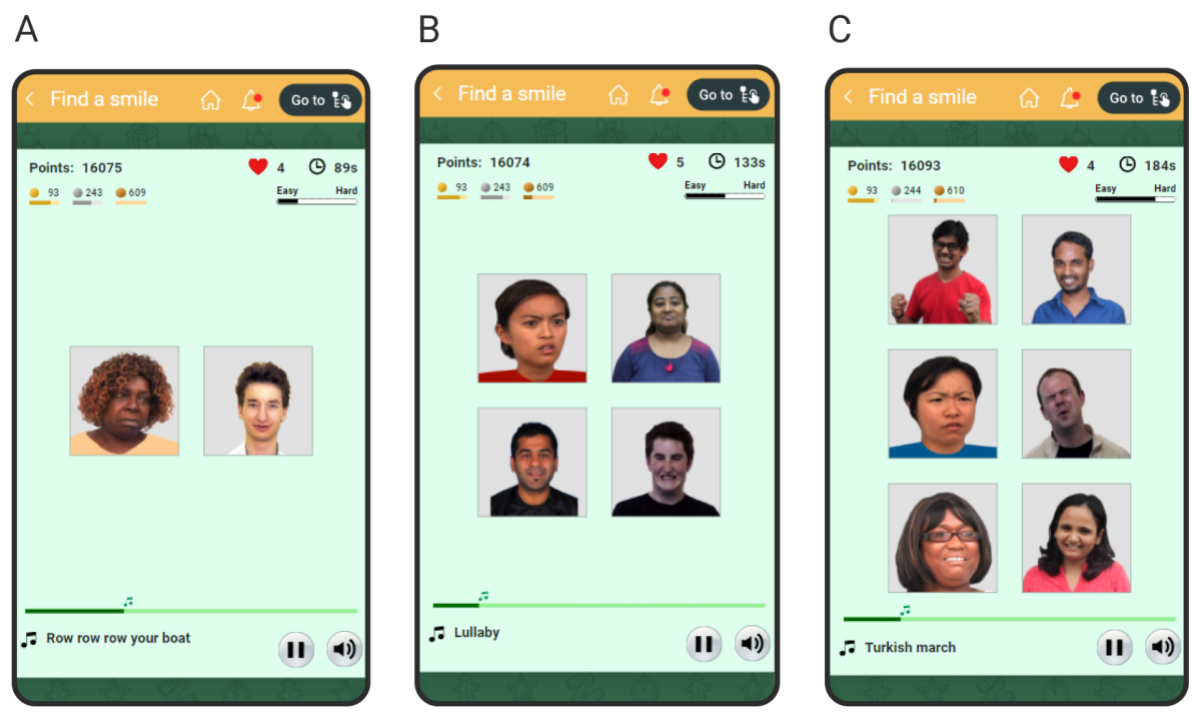
Screenshots of the game for negative attention bias training, showing (A) a 1 × 2 grid, (B) a 2 × 2 grid, and (C) a 3 × 2 grid of faces with different emotions.

We designed a game based on the same training paradigm to overcome negative attention bias [92]. Images of human faces, one depicting a positive emotion and the others depicting negative emotions, are shown in a grid (Figure 3). The user is asked to click (or tap, when played on a touchscreen device) on the image with positive emotion as quickly as possible. Once the user clicks on the positive image, the next set of images is displayed in the same grid. To make this process fun and engaging, the game plays a musical note when the user clicks on a positive image. The musical notes, played sequentially, are taken from a popular song (or rhyme), and 1 song constitutes 1 level of the game. A click on a negative image results in an unpleasant beep. Therefore, in effect, the user can play the song by clicking on the faces with positive emotions—the more accurately they select the images with positive emotions, the fewer interruptions they hear during the song.

To make the game challenging, the user is given a time limit to finish each level, calculated as *TT = N × T*, where *N* is the number of musical notes in the song and *T* is the time allotted per note (initialized to 5000 milliseconds). The game adapts to the user’s performance level by dynamically varying *T* between 1000 and 5000 milliseconds and the grid size between 1 × 2 (Figure 3A), 2 × 2 (Figure 3B), and 3 × 2 (Figure 3C) after the completion of each level. The algorithm of this adaptation is summarized in Figure S3 in Multimedia Appendix 1.

A bar, ranging from *Easy* to *Hard*, indicates the current difficulty level. To dissuade the user from clicking on images without paying attention, we penalize each click on a negative image by deducting 1 life (in addition to playing the unpleasant beeps). The user is given 5 free lives at the beginning of each game. Once the user runs out of lives, they have the option to replay the same level. Clicks on positive images earn points, which can be used to buy more time if the user runs out of time before the completion of a song. To give the user a sense of progress at each level, a progress bar on the screen indicates the fraction of notes in the current song that has been played.

The users also receive badges as short-term rewards. They earn bronze, silver, and gold badges by clicking on 34, 60, and 156 positive images, respectively. The game has 20 levels (songs). Once the user finishes all the songs, the game loops back to the first song.

### Game for Positive Imagery Training

Negative automatic thoughts can be verbal or imaginal [93]. Holmes et al [94] developed an interpretation bias training paradigm based on positive mental imagery. Multiple studies have demonstrated that the training has a positive effect on the participants’ moods [25,94-96], although there are some exceptions [36,97]; the training also helped in targeting anhedonia [97].

**Figure 4:**
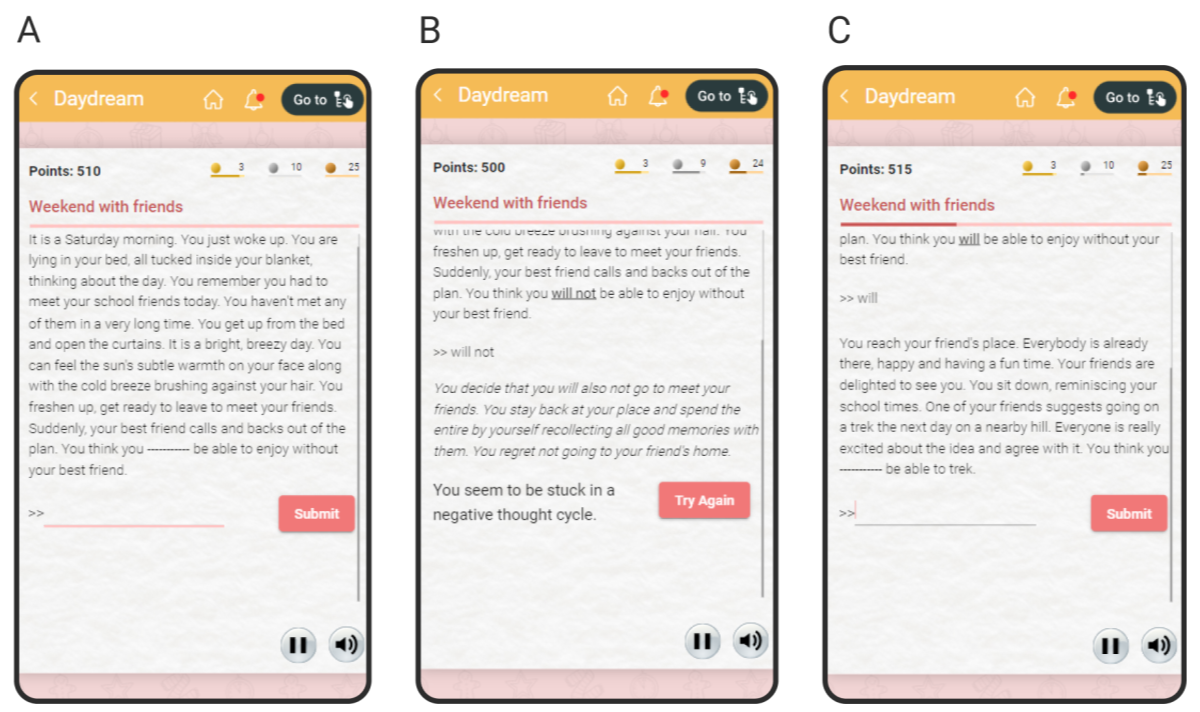
Screenshots of the game for positive imagery training. (A) A scenario is shown that can be resolved positively or negatively by providing one or more words to fill in the blank. (B) If the user resolves the scenario negatively, they are asked to try again. (C) If the user resolves the scenario positively, the game moves forward and another scenario is presented.

We designed a serious game based on the combination of paradigms by Holmes et al [94] and Mathews and Mackintosh [98]. This game was developed as interactive fiction inspired by Zork [99]. At each level, the user is presented with an ambiguous scenario in the form of a paragraph with a blank space (Figure 4A). The user can fill in the blank with one or more words to resolve the sentence positively or negatively (eg, *You think you ______ be able to enjoy the meeting* can be resolved positively by adding *will* or negatively by adding *will not*). The game evaluates the phrase written by the user by comparing it against a comprehensive list of possible positive and negative completions for each sentence (occasionally, if the phrase falls outside the list, the game responds that it could not understand the input and requests the user to write a different phrase). If the user resolves the sentence negatively, it is considered an incorrect response and the user is asked to try again (Figure 4B). If resolved positively, the game moves forward and another ambiguous scenario appears in continuation with the previous one (Figure 4C). Once the user resolves the second part of the ambiguous scenario positively, the level concludes and a new scenario appears at the next level of the game. As vivid imagination is an active element [100], users are urged to imagine the situations vividly in the first person. No hints are provided in the game; however, if the user fails to answer correctly in 3 attempts, a list of potential answers is shown from which the user can select one. To encourage vivid imagination, we did not include a time limit in this game.

The game is designed to be never-ending. It includes 20 levels, and after the completion of all the levels, the game loops back to the first level. The user earns 5 points for resolving a scenario positively. The user also earns a bronze, a silver, and a gold badge after positively resolving 4, 10, and 26 scenarios, respectively.

### Game for Learned Helplessness

Learned helplessness is a laboratory model of depression that emulates multiple aspects of clinical depression in animals [101,102]. The state of learned helplessness, reached after some experiences of inescapable aversive conditions, involves a generalized self-assumption of powerlessness, thereby reducing the effort to come out of difficult situations in the future. A reformulation of the theory in terms of attribution theory has also been extended to humans: individuals with learned helplessness attribute their failures to personal, pervasive, and persistent lack of abilities and their successes to luck [103].

We designed a puzzle-based game to help the users understand the ideas of learned helplessness and attribution style. We reasoned that an explanation after a game-based transient experience of the phenomenon would be more effective than simply explaining the concept using text or videos and is more likely to help users change their self-defeating attribution bias.

In the original experiments on learned helplessness, dogs failed to escape avoidable shocks after they had unsuccessfully tried to escape unavoidable shocks [104]. Very recently, a paradigm has been proposed to test learned helplessness in humans using loud audio tones as a stressor [105]. To allow the users to appreciate this phenomenon without a physical stressor, we devised the following approach: if a user can solve a puzzle initially but gives up on the same puzzle when it is shown after the failure to solve a hard and unrelated puzzle, the user would be able to see the parallel with learned helplessness and realize that it can happen in real life. At this point, the user is more likely to be receptive to the introspection of their own attribution bias.

We created a suite of 4 puzzle-based mini-games to achieve this objective. The first one, the circle-triangle mini-game, is a genuine puzzle game with multiple difficulty levels. The other 3 mini-games are designed to trick the user: each of them appears like a genuine puzzle game with easy puzzles in the first 1 or 2 levels but has unsolvable levels thereafter.

**Figure 5:**
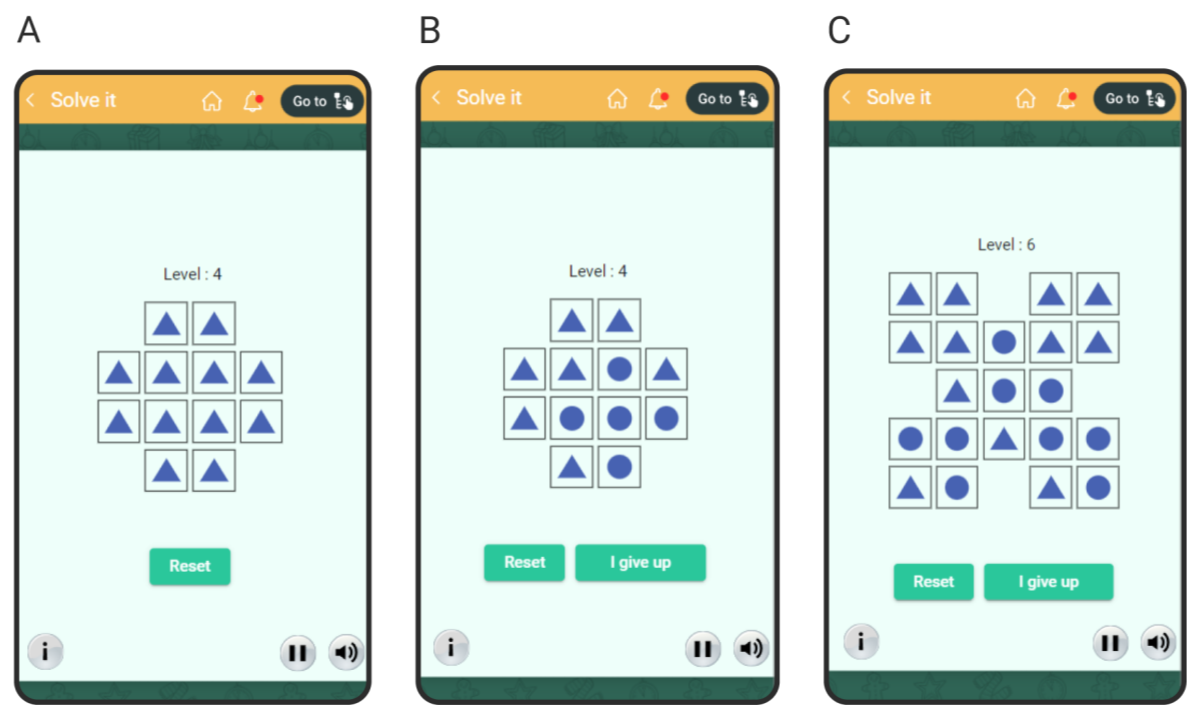
Screenshots of the circle-triangle mini-game in the game for learned helplessness. (A) The initial state in level 4. (B) An intermediate state in level 4. (C) An intermediate state in level 6.

The user starts by playing the circle-triangle mini-game. The screen presents a pattern of squares, each containing a circle or a triangle (Figure 5A). If the user clicks on one of the squares, it flips (a circle becomes a triangle and vice versa). Simultaneously, the adjacent squares also flip (Figure 5B). The goal of the user is to change all the embedded shapes into circles. Once the user solves the puzzle, a message (*Great*) and auditory feedback (a pleasant *ding*) are provided to the user. Next, a harder level of the same mini-game is presented. This stepwise increase in the level continues until the user fails to solve the current level and clicks on a button labeled *I give up* (Figure 5C). When this happens, 1 of the 3 unsolvable mini-games is displayed to the user.

**Figure 6:**
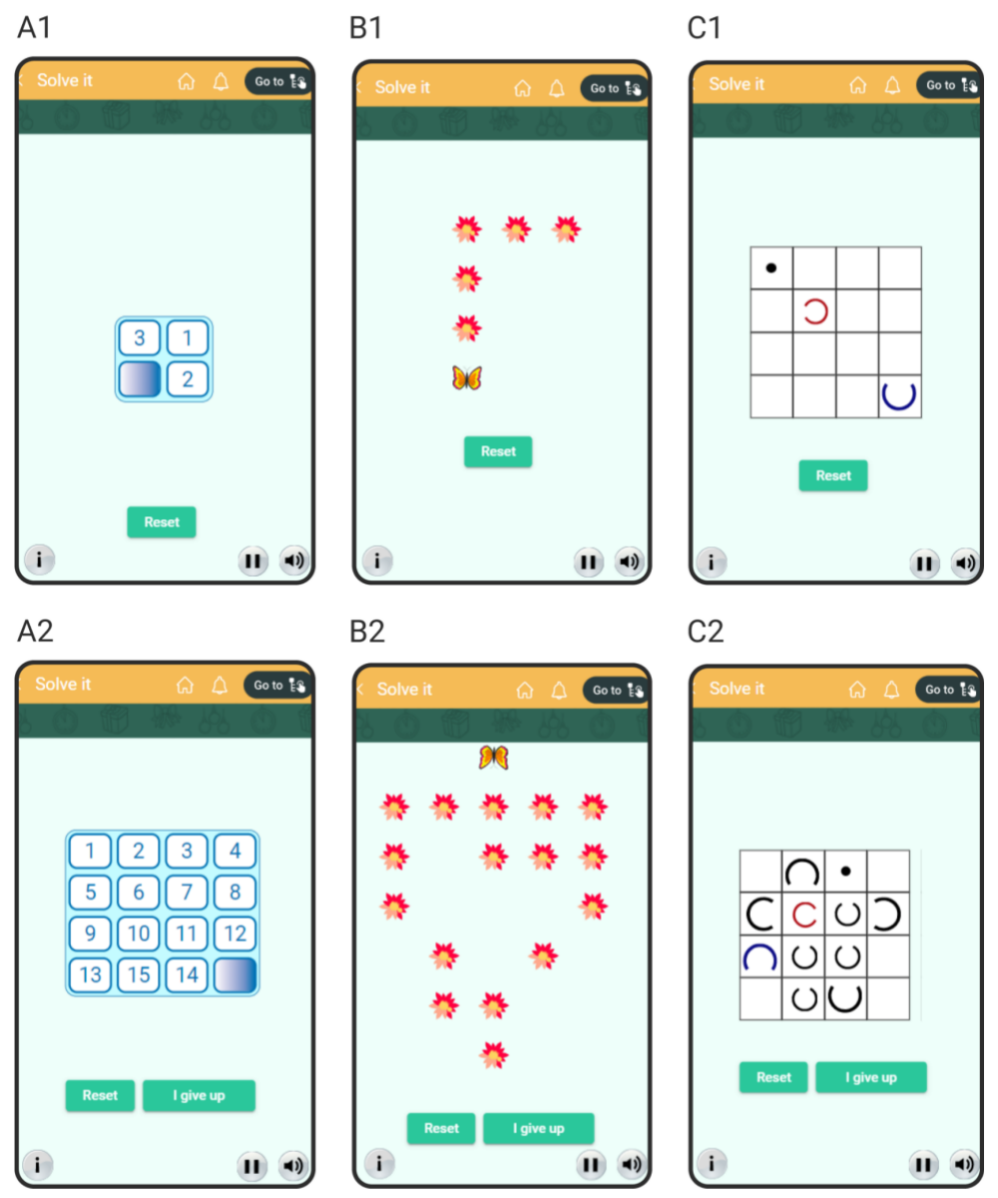
The 3 unsolvable mini-games within the game for learned helplessness. (A) The goal in this mini-game is to arrange the tiles in the increasing order of the numbers. Configuration A1 is solvable, and A2 is unsolvable. (B) The goal in this mini-game is to get the butterfly to sit on all the flowers. The butterfly can be moved forward and sideways using the cursor keys on a keyboard or the swipe action on a touchscreen device. Once a butterfly sits on a flower, the flower withers away and the butterfly cannot move to a position without a flower. B1 is solvable, and B2 is unsolvable. (C) The goal in this mini-game is to insert the small red arc into the big blue arc. The user can move the solid black ball using cursor keys or swipe action to the adjacent squares (if the square contains an arc open in the direction of the ball, the ball moves inside the arc). Once the ball is inside an arc, the arc can be moved along with the ball to an adjacent square that is empty or contains a bigger arc with an opening in the direction from which the smaller arc is coming. The ball comes out of the small arc if moved in the direction in which the arc is open. C1 is solvable, and C2 is unsolvable.

The 3 unsolvable mini-games are based on different types of puzzles (Figures 6A-C). For example, one is based on the popular *15 puzzle*, in which a 4 × 4 grid has 1 empty cell and 15 cells containing tiles numbered 1 to 15 and the goal is to move the tiles to arrange them in increasing order. The first level shown in the mini-game is kept very simple and easily solvable (Figure 6A1), which allows the users to become familiar with the puzzle. However, unbeknownst to the user, the second and third levels are unsolvable—we set the initial configuration of tiles in these levels by slightly reordering the tiles from solvable puzzles in such a way that they could not be solved anymore but looked normal otherwise (Figure 6A2). Thus, at the second level—just after the very easy first level—the user finds it impossible to solve the puzzle and has no choice but to accept failure by clicking on the button *I give up*. After this, the third level of the same mini-game appears, in which the user again gives up regardless of how hard they try.

**Figure 7:**
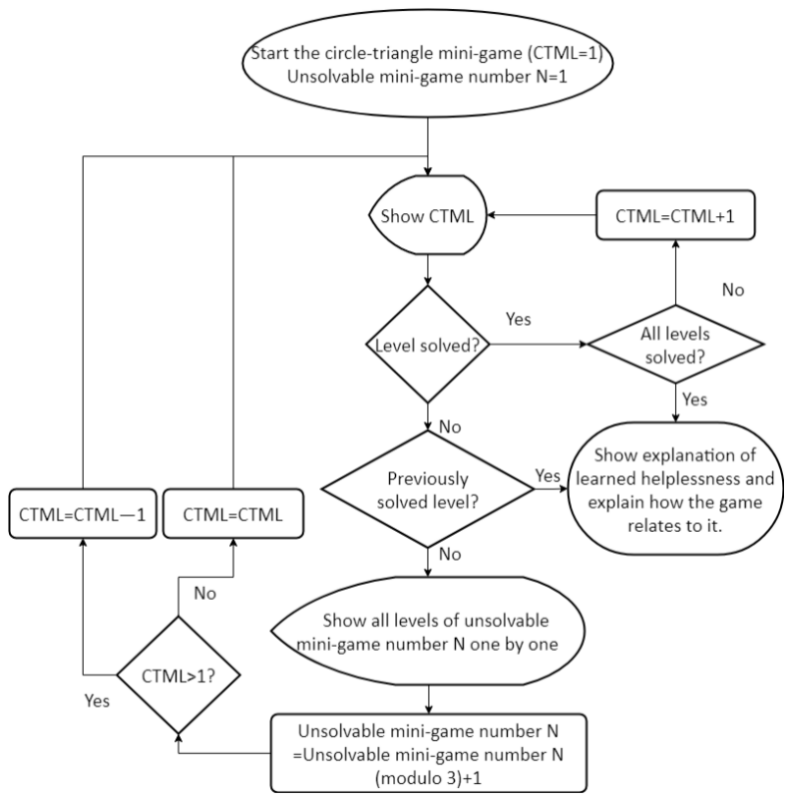
Flowchart describing the sequence of mini-games in the game for learned helplessness. CTML: circle-triangle mini-game level.

Once the user gives up in both the levels of an unsolvable mini-game, the last solved level of the circle-triangle mini-game is shown again (Figure 7 presents the flow of mini-games). If the user gives up on this level, which they have previously solved, we take the opportunity to draw a parallel with learned helplessness and the game ends with the explanation. By relating to the game, we explain the ideas of learned helplessness and attribution bias, with an emphasis on personalization and pervasiveness. We expect that once individuals can appreciate their own susceptibility, they are more likely to engage in the introspection to identify real-life situations where they fall prey to dysfunctional attribution styles. A correct understanding helps one to evaluate each situation independently and not lose motivation in all areas of life upon experiencing failure in some.

If the user does not give up on the last solved level of the circle-triangle mini-game, they reach the next level within the same mini-game, and the game continues as normal. Once they give up on a higher level of the circle-triangle mini-game, they are shown a different unsolvable mini-game. This cycle of alternating between the circle-triangle mini-game and a different unsolvable mini-game continues until the user gives up on a previously solved level of the circle-triangle mini-game or completes all levels in it (the difficulty of the higher levels ensures that the latter scenario is unlikely).

The 4 puzzle-based mini-games are designed to be engaging and challenging to play. The difficulty level in the circle-triangle mini-game adapts to the competence level of the users. The screen displays the current level to provide the user with a sense of progress. The user receives a gold badge on finishing the game. Unlike other games in TreadWill, this game was designed to be played for a limited time, until the explanation of learned helplessness and the idea of the game were shown; however, the users are free to play it again if they wish.

### How Game-Like Are Our Games?

Cugelman [106] has suggested 7 core elements of gamification. Each of the games we had designed incorporated 6 of the 7 elements, including goals, challenges, feedback, rewards, progress, and fun; the only element not included in our games is social connectivity, as the games were designed to be played individually as part of a mental health intervention. Table 1 describes how the core elements are incorporated into each game.

**Table 1:** Core game elements incorporated in different games.

Game	Goals	Challenges	Feedback	Rewards	Progress	Fun
Automatic interpretation bias modification	Finding words in the alphabet grid to complete the sentence	Finding words from the grid within the allotted time	Points for finding words and positive interpretation	Points and badges	In-level progress using progress bar In-game progress using difficulty bar	Finding words in the alphabet grid
Executive control training	To keep the avatar alive as long as possible and collect coins	Avoid the obstacles at an increasingly faster speed	Points for keeping the avatar alive	Points and badges	In-game progress via increasing avatar speed	Gameplay similar to *Super Mario Bros.*
Negative attention bias modification	To click on positive faces	To complete the song within the allotted time and avoid clicking on negative faces	Point and progress in level for clicking positive face and reduction in life for clicking on negative face	Points and badges	In-level progress using progress bar In-game progress using difficulty bar	The music along with the game play
Positive imagery training	To progress in the game by providing positive resolutions	Finding the right word	Points and progress in level for positive resolutions	Points and badges	In-level progress using progress bar	Imagining the self-referent situations
Learned helplessness	Solving the puzzles	Solving the puzzles	Positive feedback for solving a puzzle	Badge	For the solvable game, levels are shown	Solving the puzzles

### Feedback on the Games

**Figure 8:**
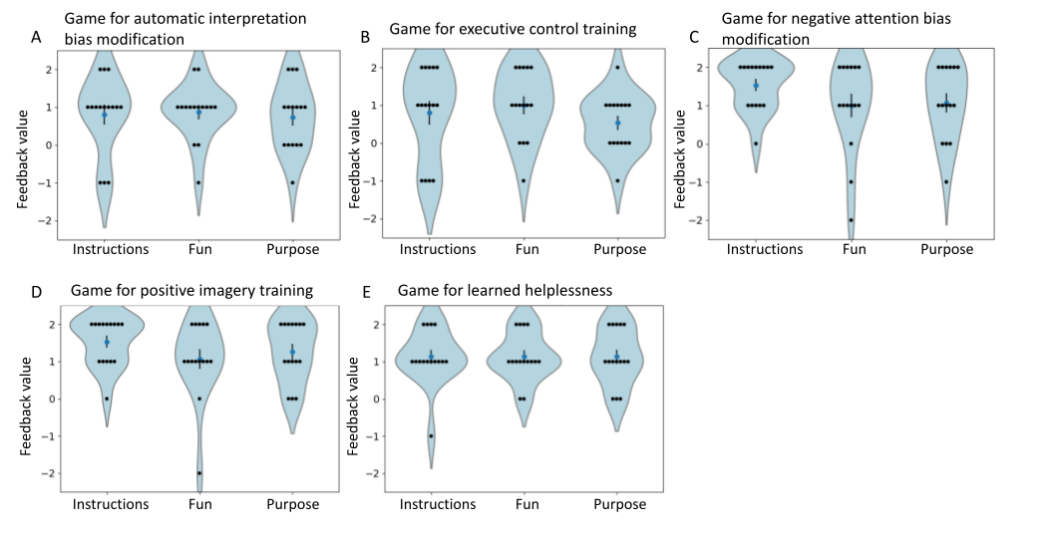
Acceptability results of the game for (A) automatic interpretation bias modification, (B) executive control training, (C) negative attention bias modification, (D) positive imagery training, and (E) learned helplessness. Overall (1) the instructions for the games were clear; (2) the games were fun to play; and (3) the purpose of the games was clear.

The feedback for all games obtained from the 15 pilot users (*Methods* section) is summarized in Figure 8. In most cases, there was statistically significant positive feedback on the clarity of instructions, fun in gameplay, and clarity of purpose (all *P*<.05; Table 2), showing that the games were acceptable to the users. According to the user feedback (Figure 8A-E), the games for negative attention bias modification (Figure 8C) and positive imagery training (Figure 8D) had the clearest instructions; the game for learned helplessness (Figure 8E) was the most fun to play; and the game for positive imagery training (Figure 8D) had the clearest purpose. The game for learned helplessness was able to achieve its purpose: 11 (73%) out of 15 users said that they had a better understanding of the idea of learned helplessness after playing the game. The game was able to induce anger or frustration in 6 (40%) out of 15 participants, of which 4 (67%) of the 6 mentioned that their anger or frustration reduced once the logic of the game was explained (overall, 7/15, 47% users reported a reduction in anger or frustration). Only 2 (13%) out of 15 participants reported that their anger or frustration did not reduce once the logic of the game was explained.

**Table 2:** Summary of feedback on the games from 15 pilot users. Each game was rated on 3 parameters on a 5-point Likert scale, in which 2=strongly agree, 1=agree, 0=neutral, −1=disagree, −2=strongly disagree. The *P* value corresponds to Wilcoxon signed rank test against 0.

			Values, mean (SD)		*P* value
**Automatic interpretation bias modification**					
	Instructions	0.800 (1.014)		.02	
	Fun	0.867 (0.743)		.003	
	Purpose	0.733 (0.883)		.02	
**Executive control training**					
	Instructions	0.800 (1.207)		.03	
	Fun	1.000 (0.926)		.004	
	Purpose	0.533 (0.743)		.04	
**Negative attention bias modification**					
	Instructions	1.533 (0.639)		<.001	
	Fun	1.000 (1.195)		.01	
	Purpose	1.067 (0.961)		.003	
**Positive imagery training**					
	Instructions	1.533 (0.639)		<.001	
	Fun	1.067 (1.032)		.005	
	Purpose	1.267 (0.798)		<.001	
**Learned helplessness**					
	Instructions	1.133 (0.743)		<.001	
	Fun	1.133 (0.639)		<.001	
	Purpose	1.133 (0.743)		<.001	

## Discussion

### Principal Findings

This paper describes the design of 4 serious games based on CBM paradigms and 1 serious game based on the learned helplessness theory. We expect that delivering the CBM paradigms in the form of serious games will increase the paradigms’ engagement and consequent effectiveness. Currently, cCBT is the predominant digital intervention modality used for depression. Despite some early attempts at combining cCBT and CBM [23,32,33], no well-evaluated and widely available software intervention offers both. One likely reason is that users are not motivated to use the CBM paradigms in their raw forms. Incorporating game-based CBM paradigms in digital interventions might serve the following 2 purposes: increase engagement with the overall interventions and serve as a complementary therapeutic approach to CBT. Overall, all the games received positive feedback on all 3 aspects—whether the instructions were clear, whether the game was fun to play, and whether the purpose of the game was clear (Figure 8). In addition, the learned helplessness game was able to achieve its purpose of explaining learned helplessness via experiential learning.

### Optimization of Game Paradigms

Two of the CBM paradigms described in this paper, the negative attention bias training [92] and the positive imagery training [94], were previously converted into serious games. In particular, the negative attention bias training paradigm has inspired many serious games. However, only a few have been evaluated in research studies [62,107,108], whereas the rest are commercially available without evaluation. The Bias Bluster game [109] was developed based on the positive imagery training paradigm.

We made slight modifications to 3 of the CBM paradigms to make them suitable for use in the games while keeping their active ingredients intact. In the original automatic interpretation bias modification paradigm, a sentence was shown for 1000 milliseconds [21]. However, during our internal testing, we observed that 1000 milliseconds was not appropriate for all sentences and, in general, too short for nonnative English speakers. Therefore, we showed the sentence for a duration dependent upon the length of the sentence, computed by *100 + (TC / ARS)* milliseconds, where *TC* denotes the total number of characters in the sentence and *ARS* denotes the average reading speed (estimated to be approximately 50 characters per 1000 ms).

In the original negative attention bias training paradigm, the images were presented in a 4 × 4 grid [92]. During the internal testing, we observed that a 4 × 4 grid on a smartphone screen makes the individual images very small and the emotions in the images indiscernible. Considering the higher penetration of smartphones than computers or tablets, we presented images in 2 × 1, 2 × 2, or 3 × 2 grids to ensure that the images and emotions could be seen easily.

In some of the previous implementations of the positive imagery training paradigms, the scenarios were presented in an auditory format and the positive resolutions were provided to the user by the program directly [25,94,95,97,100]. We presented the scenarios using a text-based format, similar to Mathews and Mackintosh [98], as the auditory format is less amenable to gameplay (eg, an audio clip is slower to scan back and forth compared with text). Furthermore, we designed the game such that the user was required to come up with a positive resolution to the ambiguous scenario. This modification was inspired by the observation that the training was more effective in changing the mood if the interpretation was actively generated by the user [98].

### Limitations and Future Directions

Feedback from the users in this study indicates that serious games based on CBM paradigms are acceptable. However, this study had a small sample size. There is a need for more studies with larger, more diverse, and clinical samples to test the real-world engagement and effectiveness of the games. In the future, games can be made more engaging using virtual reality.

### Conclusions

Serious game–based mental health interventions are acceptable to users and have the potential to increase their engagement with digital mental health interventions. The designs we have provided can also be adapted into other interventions for many other disorders in which cognitive biases are involved [110]. We expect that the inclusion of serious games based on the CBM paradigms will help in improving the effectiveness and engagement of digital interventions.

## Appendix

Multimedia Appendix 1 Flowcharts describing game algorithms.

## Abbreviations

CBM: cognitive bias modification
CBT: cognitive behavioral therapy
cCBT: computerized cognitive behavioral therapy

## Data Availability

The deidentified data analyzed in the pilot study are available from the corresponding author upon reasonable request.
