# Supplementary material for: Serious Games Based on Cognitive Bias Modification and Learned Helplessness Paradigms for the Treatment of Depression: Design and Acceptability Study
Source: JMIR Serious Games. 2023 May 3;11:e37105. doi: 10.2196/37105 (PMC10193222; doi:10.2196/37105)
Supplement: Multimedia Appendix 1 [file games_v11i1e37105_app1.pdf]

Serious Games Based on Cognitive Bias Modification and Learned Helplessness Paradigms for Treatment of Depression: a Design and Acceptability Study.

Supplementary figures S1-S3.

Supplementary Figure S1

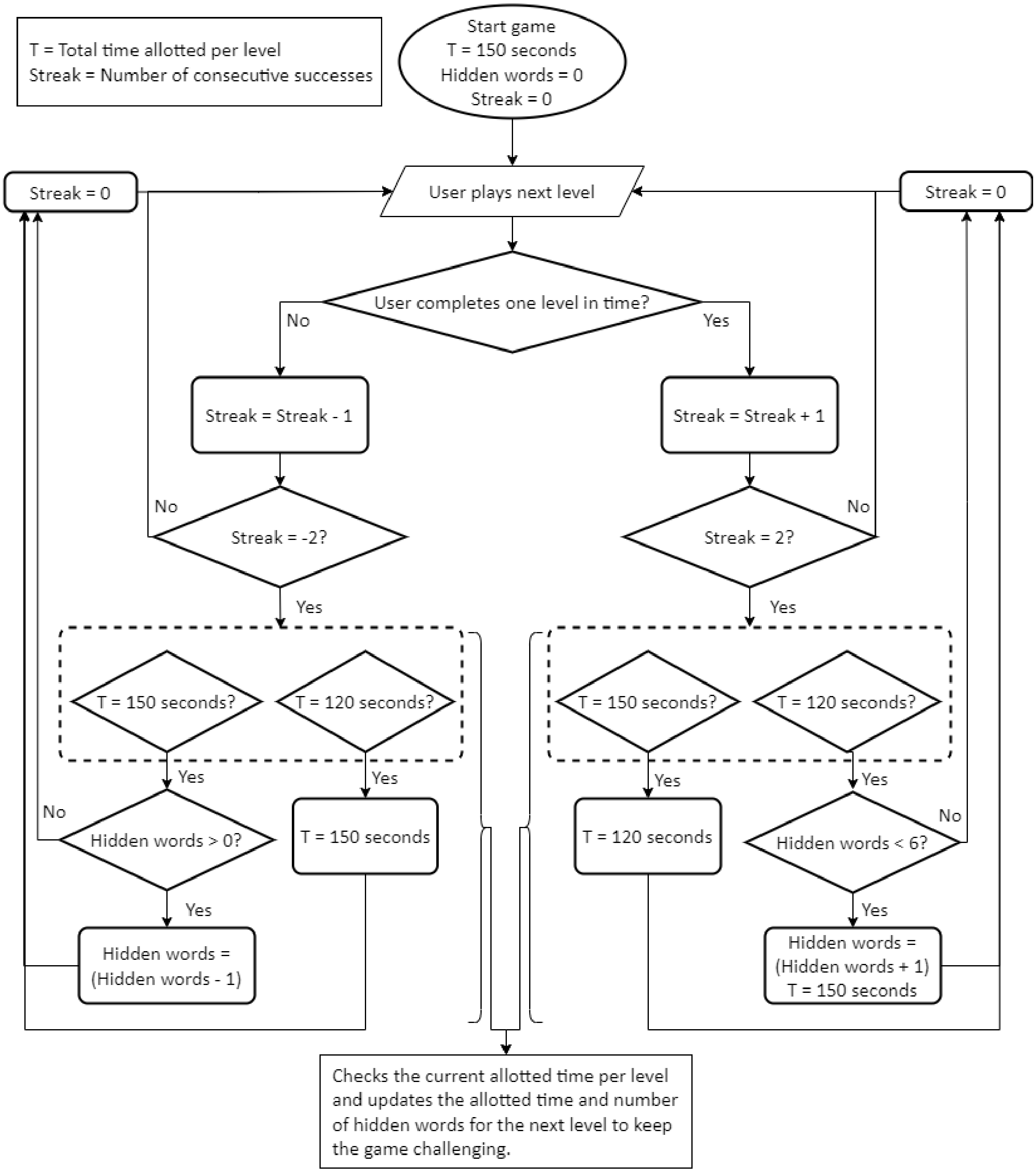

Supplementary Figure S1: Flow chart describing the algorithm for the adaptive difficulty of the game for automatic interpretation bias modification.

Supplementary Figure S2

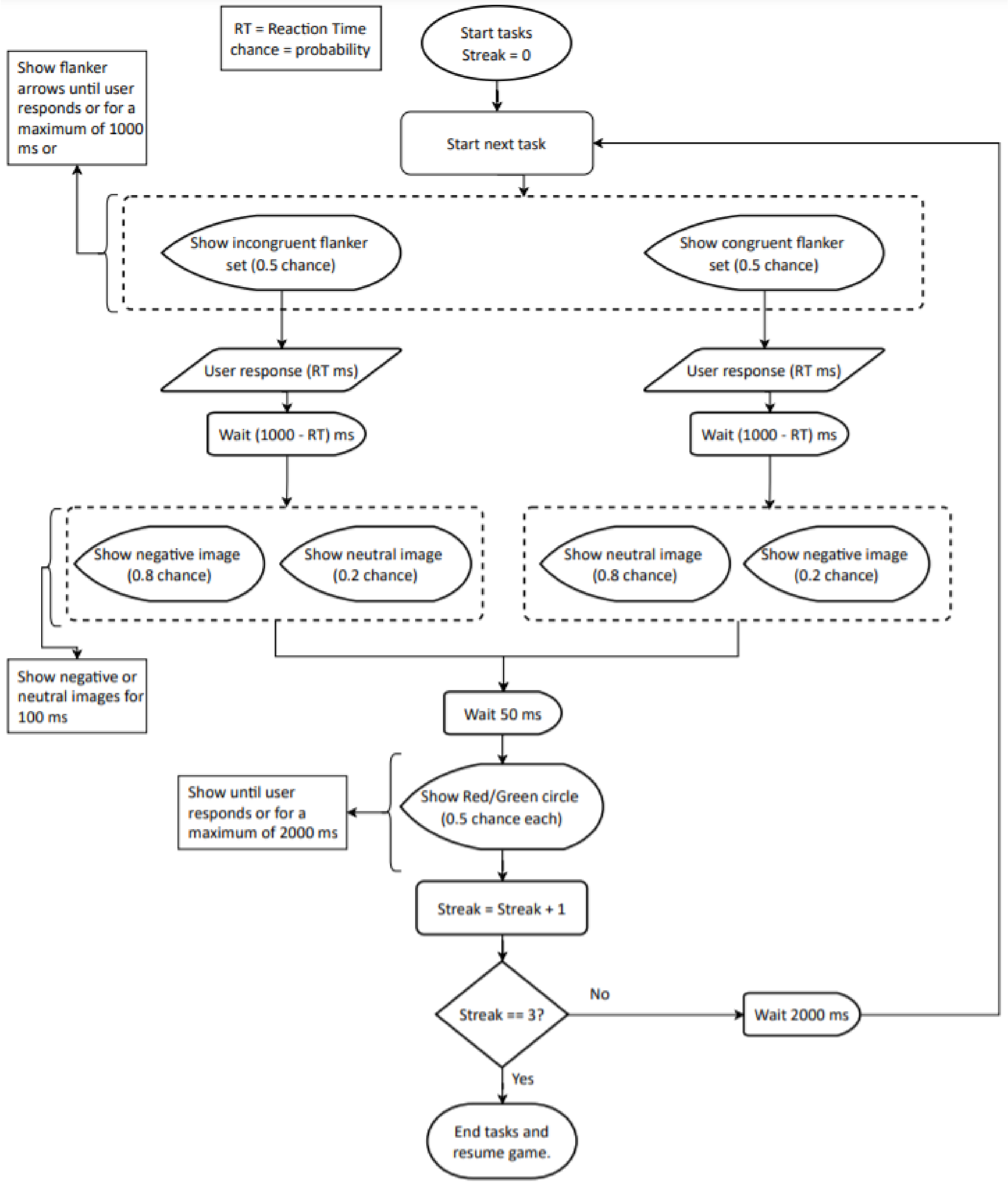

Supplementary Figure S2: Flow chart describing the CBM paradigm used in the game for executive control training.

Supplementary Figure S3

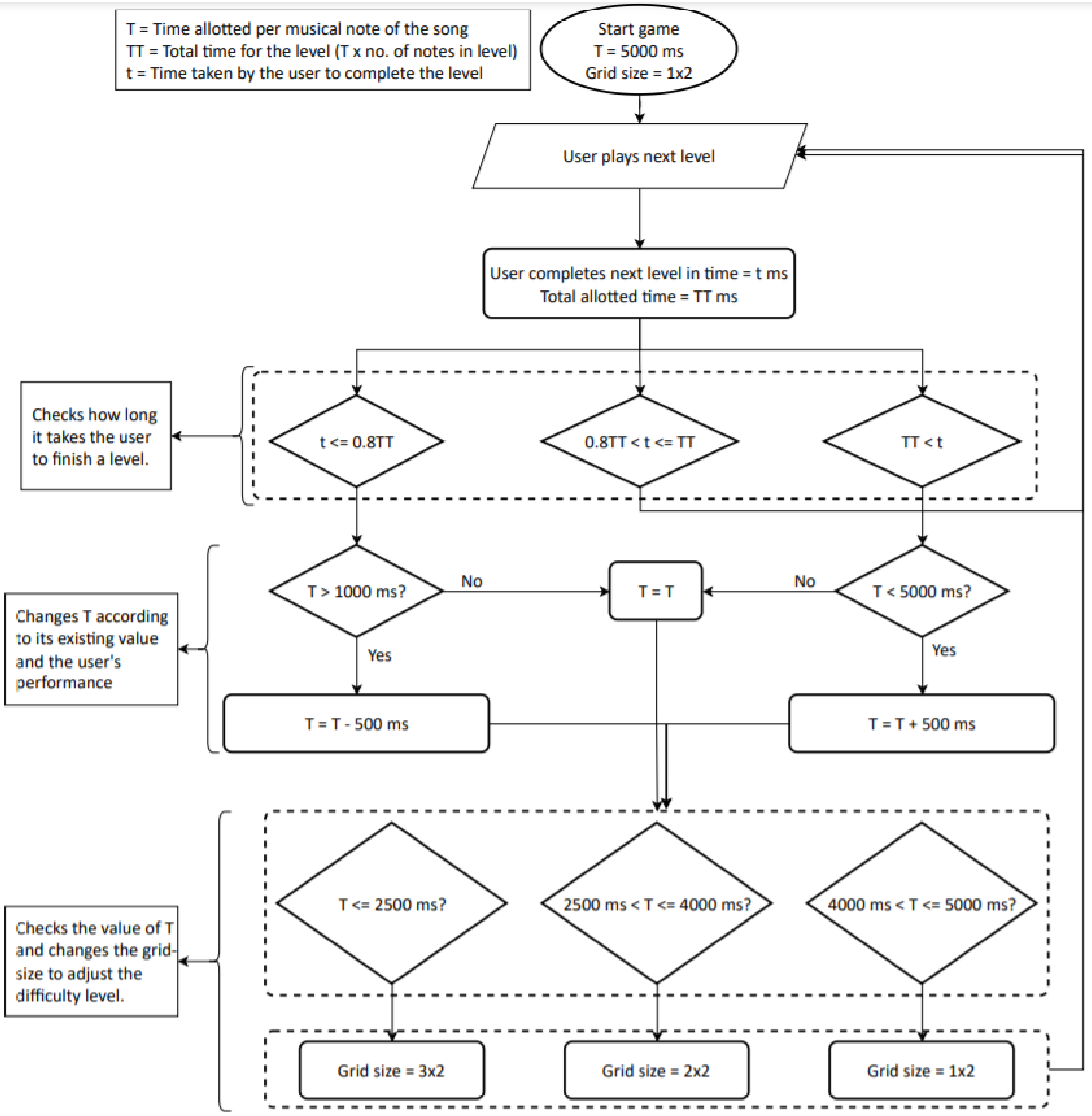

Supplementary Figure S3: Flow chart describing the algorithm for the adaptive difficulty of the game for negative attention bias training.
